# A Survey of Traumatic Brain Injuries from Road Traffic Collisions in a Lower Middle-Income Country

**DOI:** 10.7759/cureus.36892

**Published:** 2023-03-29

**Authors:** Muhammad Tariq Barki, Faiqa Filza, Almas F Khattak, Osama Bin Khalid, Mustafa Qazi, Humaira Gilani, Shahid Ayub, Muhammad Farooq

**Affiliations:** 1 Neurosurgery, Northwest General Hospital and Research Center, Peshawar, PAK; 2 Community Medicine and Research, Northwest School of Medicine, Peshawar, PAK; 3 Medicine and Surgery, Northwest School of Medicine, Peshawar, PAK; 4 Dermatology, Northwest General Hospital and Research Center, Peshawar, PAK; 5 Neurosurgery, Hayatabad Medical Complex Peshawar, Peshawar, PAK; 6 Neurosurgery, Khyber Girls Medical College, Peshawar, PAK; 7 Neurosurgery, Afridi Medical Complex and Teaching Hospital, Peshawar, PAK

**Keywords:** neuro-rehabilitation, pre-hospital emergency, prevention, survey, neurotrauma in low income countries, lower middle income country, road traffic collisions, traumatic brain injury, tbi, traumatic injury

## Abstract

The burden of traumatic brain injury (TBI) from road traffic collisions (RTCs) is great in low-and middle-income countries (LMICs) due to shortfalls in preventative measures, and the lack of relevant, accurate data collection. To address this gap, we sought to study the epidemiology of TBI from RTCs in two LMIC neurosurgical centres in order to identify factors amenable to preventative strategies. A prospective survey of all adult and paediatric cases of TBI from RTCs admitted to Northwest General Hospital (NWGH) and Hayatabad Medical Complex (HMC) over a four-week period was carried out. Data on patient demographics, risk factors, injury details, pre-hospitalisation details, admission details and post-acute care was collected and analysed. A total of 68 patients were included in the study. 18 (26%) of the patients were male and in the 30 to 39 age group. Fifty-two percent were two-wheeler riders and/or passengers. 51 (75%) of the RTCs occurred between 12 noon and 12 midnight and in rural areas (66.2%). The most commonly documented risk factor that led to the RTC was speeding (35.3%). Pre-hospital care was either absent or undocumented. Up to two-thirds of patients were not direct transfers, and most were transported in private vehicles (48.5%) arriving later than an hour after injury (94.1%). Less than half with documented disabilities were referred for rehabilitation (38.5%). There are still gaps in the prevention of TBI from RTCs and in relevant data collection. Data collection systems must be strengthened, and further exploratory research carried out in order to improve the prevention of TBI from RTCs.

## Introduction

Traumatic brain injury (TBI) is a significant issue in the health world [1]. According to recent studies, there are 500 to 800 new cases of TBI per 100,000 people, accounting for up to 50% of all trauma-related deaths, and 15 to 20 people with disabilities for every 100,000 people [2,3].

One of the most important causes of TBI seen worldwide is road traffic collisions (RTCs) where the burden is greatest in low-and middle-income countries (LMICs) [1-3]. Rapid motorisation and urbanisation without appropriate safeguarding measures have put many LMIC populations at an increased risk of RTCs [3,4]. Along with infrastructure, environmental, resource, and population issues, these nations frequently struggle with inadequate road and vehicle engineering, lax enforcement, a lack of targeted education, and a lack of knowledge regarding road safety [5].

LMICs also lack formal and effective pre-hospital care systems with uncoordinated ambulance services [6,7]. A well-organised pre-hospital care system is essential for lowering morbidity and mortality from neurotrauma when on-scene stabilisation and quick transport are required to avoid or minimise secondary insults that could cause subsequent brain injury [6,8].

Many patients benefit from post-acute treatment or rehabilitation for moderate-to-severe TBI since it lessens the long-term effects of brain damage and lowers the risk of complications such as pressure ulcers, which have been described as still being highly incidental in neurosurgical wards [9-11]. In LMICs, these facilities are either absent or not readily available and many TBI patients do not receive rehabilitation after discharge from the hospital [12]. 

Accurate epidemiological data collection is instrumental in enabling an objective understanding of these challenges and identifying factors amenable to prevention in order for the appropriate strategies to be put in place [6]. While the number of studies on TBI is growing in LMICs, very few illustrate the correlation with RTCs alone, despite this being the largest cause of TBI in these contexts.

In an effort to address these gaps, we carried out a survey on TBI from RTCs in two centres, i.e., Northwest General Hospital and Research Center (NWGH) and Hayatabad Medical Complex (HMC). The aim of this study is to study the distribution and determinants of TBI from RTCs, to characterise the management of patients during the pre-hospital period, and to ascertain if post-acute care was offered and the nature of this care.

## Materials and methods

Data were collected prospectively over a four-week period in two centres, i.e., Northwest General Hospital and Research Center (NWGH) and Hayatabad Medical Complex (HMC). Both study sites were the centres where all the patients with moderate-to-severe TBIs received definitive neurosurgical treatment, or ‘treatment centres’. The terms ‘study sites’ and ‘treatment centres’ will be used interchangeably in this paper.

All adult and child patients (taken as those under 18 years old) with TBI from RTCs who were admitted through the accident and emergency department of the study sites were included in the study. This encompassed all cases of mild, moderate and severe TBI, based on the Glasgow Coma Scale (GCS). The diagnostic criteria for TBI used in this study were derived from the International Classification of Disease 10th (ICD-10) and 11th Revision (ICD-11). As both centres do not use ICD codes for diagnosis, the on-call neurosurgeons ensured that the final diagnosis in the patient’s notes met the inclusion criteria.

Data on patient demographics, risk factor information, medical history, injury details, pre-hospitalisation details, and admission details were obtained from the emergency department and neurosurgical ward records at the study sites, and where applicable and necessary, from admission documents of the healthcare centres the patients were first transferred to. For cases where the pre-admission data was not recorded, interviews were carried out with patients or their relatives. Data on post-acute care were collected at four weeks, discharge or death, whichever came first. This was sourced from the neurosurgical ward records. All data was first recorded on paper questionnaires which had been pre-tested and then transferred onto a Microsoft ® Excel spreadsheet. As a way of maintaining participant confidentiality, patients were identified by numbers.

IBM® SPSS® version 24.0 was used to analyse the data. Descriptive statistics were used to summarise both continuous and discrete variables as frequencies and percentages.

## Results

Demographics and road user type

A total of 68 patients were admitted to both study sites over the study period. Three-quarters of patients were male and 18 (26.4%) of the injuries occurred in the 30 to 39 age group. 52 cases were reported from the Hayatabad Medical Complex, while 16 cases were reported from the Northwest General Hospital and Research Centre (NWGH & RC). Three-quarters of patients were male and most of the injuries occurred in the 30 to 39 age group (Table 1).

**Table 1:** Socio-demographic characteristics of patients

Parameters	Total, N (%)
Age (years)	
0-9	4 (5.9)
10-19	9 (13.2)
20-29	11 (16.2)
30-39	18 (26.4)
40-49	12 (17.6)
50-59	11 (16.2)
>59	3 (4.5)
Gender	
Male	51 (75)
Female	17 (25)
Occupation	
Students	12 (17.6)
Manual worker (i.e. labourer, farmer, factory worker)	34 (50)
Skilled worker (i.e. electrician, plumber, tailor, secretary)	6 (8.8)
Professional (i.e. lawyer, doctor, engineer)	1 (1.5)
Unemployed	14 (20.6)
Not applicable	1 (1.5)
Category of road user	
Pedestrian	19 (27.9)
Two-wheeler riders and/or passengers	36 (52.9)
Four-wheeler drivers and/or passengers	10 (14.7)
Three-wheeler drivers or passengers	1 (1.5)
Unknown	2 (3.0)

Medical history

Majority of patients did not have any known medical history (86.7%). Sixteen patients smoked cigarettes regularly, whereas only three abused other drugs. None of the patients had a history of TBI.

Time and place of injury

More than three-quarters of RTCs occurred between 12.01 pm and midnight with almost equal numbers occurring between 12.01 and 6 pm, and 6.01 pm and 12 am (Table 2).

**Table 2:** Time of RTCs

Time	Total, N (%)
12.01 AM-6 AM	4 (5.9)
6.01 AM-12 noon	13 (19.1)
12.01 PM-6 PM	26 (38.2)
6.01 PM-12 AM	25 (36.8)

Factors and risk factors leading to RTCs and related TBI

Human factors appeared to be the leading cause of RTCs, where speeding was the most common risk factor recorded. However, in about a third of patients, the cause was unknown (Table 3).

**Table 3:** Factors and risk factors leading to RTCs

Factors		Total, N (%)
Human Factors	Speeding	24 (35.3)
	Jaywalking	2 (3)
	Drink driving	3 (4.4)
	Not using conspicuity equipment	1 (1.5)
Environmental Factors	Collision with animals suddenly emerging onto road	3 (4.4)
	Poor road conditions	3 (4.4)
	Fault of opposite driver	10 (14.7)
Unknown		22 (32.3)

Only two patients (5.6%) who were either riders or passengers on two-wheelers reported using helmets. Similarly, only one out of 10 patients who were four-wheeler drivers or passengers reported using a seatbelt. In two cases (4.3%), the use of these personal protective equipment was not known.

Transport and pre-hospital care

The time taken for the ambulance call to be made was recorded in 18 cases, where most calls were made within the first 10 to 14 minutes of the RTC. Ambulance arrival was mostly within 15 to 39 minutes of the call. In none of the RTCs did the ambulance arrive in less than 10 minutes of the call being made (Table 4).

**Table 4:** Mode of transport to treatment centres

Mode of transport	Nature of transfer to treatment centres		Total (%)
	Direct transfer	Referral	
Ambulance	13	19	32 (47.0)
Private vehicle	8	25	33 (48.5)
Police car	-	1	1 (1.5)
Public transportation	-	1	1 (1.5)
Unknown	1	-	1 (1.5)
Total (%)	22 (33.8)	46 (66.2)	

Although paramedics and medical personnel were the main group accompanying the patients transported by ambulance to study site 1 (69.6%), in study site 2, most were accompanied by family members (90%). For patients arriving in private vehicles, majority were brought in by family members (84.8%). Two were brought in by law enforcement officials (6.1%), and three by passers-by (9.1%). The single patient who arrived with public transportation (auto-rickshaw) was brought in by a passer-by.

There were no records of any emergency pre-hospital care at the scene, and no records of the patients transported by ambulance receiving any assessment of vital signs, level of consciousness or pupillary response to light, nor any pre-hospital care delivered en-route. Further investigation by local researchers indicated that no pre-hospital care was provided for more than three-quarter of patients (n=52, 76.5%), even those attended to and transported by emergency medical services (EMS).

Three patients were attended by bystanders and two by paramedical staff and provided some form of prehospital assistance, although the nature of this was not recorded. The provision of pre-hospital care was unknown in 19.1% of cases.

Records of treatment received at the other healthcare centres were obtained only from study site 1. Most healthcare centres (82.9%) provided some form of treatment which ranged from primary care to advanced life support. Only four centres did not provide any treatment, all of which were Primary Healthcare centres.

Distance of travel and time taken to arrive at treatment centre

Most RTCs occurred less than 50 kilometres from the study sites, where 14 took place less than 10 kilometres from the treatment centres (48.3%).

Only four patients arrived in less than 60 minutes of the injury, all of which were direct transfers to the treatment centres. Most patients were admitted between 1 and 5 hours, and 5 to 10 hours of injury where 19 (46.3%) were direct transfers to the study sites. Twenty-three patients were admitted 10 hours or more after the RTC where more than half (56.5%) were referred from other healthcare centres.

Clinical findings on admission, management and outcomes

Most of the patients had on admission, before resuscitation, a Glasgow Coma Scale (GCS) score of between 3 and 8, followed by a GCS score of between 13 and 15 (Table 5).

**Table 5:** Clinical findings on admission and outcomes

Parameters	Total (%)
Glasgow Coma Scale (GCS) on arrival to treatment centre	
13-15 (mild TBI)	24 (35.3)
9-12 (moderate TBI)	17 (25)
3-8 (severe TBI)	27 (39.7)
Pupillary response	
Normal (bilateral reactive)	58 (85.3)
Bilateral non-reactive	4 (5.9)
Unilateral non-reactive	5 (7.3)
Unknown	1 (1.5)
Management	
Surgical	9 (13.2)
Non-surgical	59 (86.8)
Outcome within 4 weeks	
Alive	45 (66.2)
Dead	23 (33.8)
Documented Glasgow Outcome Scale within 4 weeks 5 4 3 2 1	33 (48.5) 7 (10.3) 5 (7.4) - 23 (33.8)
Disabilities at discharge (n=45)	
None	31 (68.9)
Mobility/physical	8 (17.8)
Cognitive/psychological	5 (11.1)
Unknown	1 (2.2)

Majority of patients did not receive any surgical management (Table 5). This was either because they died before arriving at the facility (78.3%), or the injury did not require surgery, or was not amenable to surgery.

Mortality was calculated at 33.8%, where 74.1% of those with severe TBI died within four weeks of admission to the study sites. Almost all these patients were referrals from other healthcare centres (92.6%) and more than half admitted 5 hours or more after the RTC (58.6%). The remaining deaths were from patients with moderate head injury, most of whom were managed surgically (67.7%). All patients with mild TBI survived and were discharged home within four weeks.

Among those who were alive at four weeks or discharge, if that came earlier, most were reported as having no disability (Table 5). For those with documented disabilities, judged according to the Functional Independence Measure (FIM) Scale, five (83.3%) from study site 1 received rehabilitation upon discharge and were referred to the hospital’s rehabilitation services whereas none from study site 2 received any rehabilitation, regardless of whether they were discharged home or to a district hospital. The details of patients from site 1 who received rehabilitation after discharge are given in Table 6.

**Table 6:** Rehabilitation of patients from Site 1

Patient	Type of rehabilitation	Distance from home (km)	Frequency	Mode of transport
1	Physiotherapy	20	Daily	Private vehicle
2	Psychological services	120	Three times a week	Public transport
3	Psychological services	15	Three times a week	Private vehicle
4	Physiotherapy	4	Twice a week	Private vehicle
5	Physiotherapy	100	Daily	Private vehicle

## Discussion

Our study shows that most RTCs occur in males between the ages of 20 and 59 years old. This corresponds to findings from the literature where this group is more at risk for RTCs, especially in LMICs [5]. This could be due to pre-existing gender disparities in economic opportunities where men in this age group are the ones expected to work or seek work in order to support their families, which in turn leads to higher road use in this group [3,4].

Low socio-economic status is another well-documented factor that is seen among those experiencing RTCs [13,14]. This was seen in our results where most patients were manual workers, and the majority were two-wheeler riders or passengers. Two-wheelers, whether motorised or non-motorised are the most common type of vehicle in LMICs as they are the cheaper and more widely available form of transportation in these countries [15]. Other epidemiological studies from India and Pakistan also show that RTCs occur more often in this group of road users, with the most common injury being TBIs [16-20]. The vulnerability to RTCs with a greater risk of sustaining a TBI arises because they are generally less protected than four-wheeler occupants where the current infrastructure, especially in LMICs, tends to prioritise cars and other motorised four-wheelers [21]. Being from a lower socioeconomic group, there is a tendency to purchase and use low-quality second-hand vehicles with feeble headlights, failing breaks and worn tires [22]. Many would also tend to ride without helmets, as seen in our study where less than 6% of the two-wheeler riders and passengers were recorded as using helmets at the time of the RTC [22,23]. This finding was also seen in other LMIC papers where helmet use among two-wheeler riders involved in RTCs was low, even lower than 5% in one study [18]. In addition, this group is inclined to use poor-quality helmets, fails to use conspicuity equipment, and is often seen travelling with three or more people in the vehicle [22-24]. 

A few papers from India and Pakistan have reported that speeding is one of the most common causes of RTCs in these countries [20,25,26]. In LMICs, this is believed to be attributed to the absence of physical factors such as the imposition of speed limits, enforcement of laws, and speed-reducing measures such as speed bumps on roads or speed governors in vehicles [27]. However, the literature does not provide an explanation for the reason behind this risk-taking behaviour.

The timing of RTCs is important as this helps identify other risk factors that could contribute to the collisions. Although the currently available epidemiological studies from LMICs do not often study this, a review by Odero et al. reported that in LMICs, 60% to 80% of traffic injuries occurred during the day whereas a third occurred at night, particularly between the hours of 6 pm and 12 midnight [28]. Although there were some similarities with these studies, our findings showed that overall, most RTCs occurred between 12 noon and 12 midnight.

In terms of the location of RTCs, our results showed that while overall, most took place in rural areas, three-quarters of the cases admitted to Study Site 2 experienced the RTC in an urban area. The pattern of RTCs in LMICs is variable, where differences in urban-rural population distribution and migration, and infrastructure could explain why more RTCs occur in rural or urban areas [28,29].

The findings on pre-hospital care highlight the absence of a formalised system for managing trauma from the time of injury until definitive treatment. Our results showed that, overall, RTC casualties did not receive any pre-hospital care, and even where they were attended to, the type of care was not documented, and in other cases, was unknown. This was even when the ambulance service was utilised, where there was no documentation of vital signs and GCS, and no evidence of resuscitation or any pre-hospital care delivered to the casualties.

Although many casualties were transported by ambulances, most were brought into the treatment centres by private vehicles, accompanied by family members. This is not an uncommon scenario in LMICs, where other studies have shown that taxis are usually the most common mode of transportation to the hospital, especially after an RTC [19,30]. In our study, this was also the case for inter-hospital transfers, which traditionally is done by ambulances.

Despite most of the RTCs occurring within 100 kilometres of the treatment centres, most of the casualties arrived more than 5 hours after the incident. From our study, this could be attributed to delays in transportation, in particular those who were referred cases. Our results show that most of the casualties were first transported to other healthcare centres where they may have remained for a while. These centres are not equipped to manage TBI, and as a result, there would have been a delay in definitive treatment.

The rehabilitation services in LMICs are poorly organised, not well documented, and tend to be influenced by availability, access and awareness of services among both healthcare professionals and members of the public.

Limitations

Our study has several limitations. The most apparent was the number of patients included. The small sample size could potentially affect the interpretation of findings. Relating to this, our study was limited to two hospitals and as a result, it would be difficult to generalise our findings to other LMIC populations due to geographical, resource, policy and socio-cultural differences, even within the same country.

Another limitation was the presence of ‘unknown’ data, the most significant being pre-hospital care received by the casualties. This occurred due to the absence of a system within the EMS for documenting pre-hospital care as well as the fact that in-hospital records are not linked with any out-of-hospital records, including those from other healthcare centres. Also, data on loss of consciousness (LOC), alteration of consciousness (AOC), post traumatic amnesia (PTA) and associated injuries such as long bone injuries were not available for the study. 

## Conclusions

Our study was useful in identifying further steps that can be taken to improve the prevention of RTCs and resultant TBI. Data on TBI from RTCs should be collected regularly and consistently using a standardised data collection system that documents a range of epidemiological parameters. This would include the documentation of any care delivered in the pre-hospital and post-acute periods as well as information on each level of TBI prevention. Having a TBI surveillance or registry system will enable the identification of current needs which in turn will facilitate resource allocation and guide the development of appropriate interventions to address these needs. Any data collection exercise requires staff training in order to ensure that data is collected accurately and correctly. In addition, the quality of the data needs to be assessed periodically through regular checks and audits. Further research needs to be done to uncover why RTCs still occur and why there are gaps in pre-hospital and post-acute care. One of the ways this can be achieved is by identifying and quantifying current preventative strategies that are being implemented, and objectively determine the effectiveness of these approaches by way of experimental or observational studies.
